# Hydrolyzed protein formula improves the nutritional tolerance by increasing intestinal development and altering cecal microbiota in low-birth-weight piglets

**DOI:** 10.3389/fnut.2024.1439110

**Published:** 2024-10-28

**Authors:** Miaomiao Bai, Hongnan Liu, Yalu Yan, Sufang Duan, Ignatius Man-Yau Szeto, Jian He, Jinjie Hu, Yawei Fu, Kang Xu, Xia Xiong

**Affiliations:** ^1^Laboratory of Animal Nutritional Physiology and Metabolic Process, Key Laboratory of Agro-ecological Processes in Subtropical Region, Institute of Subtropical Agriculture, Chinese Academy of Sciences, Changsha, Hunan, China; ^2^Inner Mongolia Yili Industrial Group, Co. Ltd, Yili Maternal and Infant Nutrition Institute (YMINI), Beijing, China; ^3^Inner Mongolia Dairy Technology Research Institute Co. Ltd, Hohhot, China; ^4^National Center of Technology Innovation for Dairy, Hohhot, China; ^5^College of Animal Science and Technology, Hunan Agricultural University, Changsha, China; ^6^Hunan Provincial Key Laboratory of the Traditional Chinese Medicine Agricultural Biogenomics, Changsha Medical University, Hunan, Changsha, China

**Keywords:** prematurity or low birth weight, hydrolyzed protein formula, amino acid metabolism, intestinal development, gut microbiota

## Abstract

**Background:**

Prematurity or low birth weight (LBW), poses a significant challenge in global health. Exploring appropriate and effective nutritional interventions is crucial for the growth and development of LBW infants. Hydrolyzed protein formula has been suggested as a potential solution to prevent intestinal dysfunction and improve digestion and absorption in infants.

**Objectives:**

This study aimed to investigate the benefits of hydrolyzed protein formula on feeding intolerance, intestinal morphological development, and microbiota in a LBW piglet model.

**Methods:**

A total of 24 male piglets (3 d of age, 0.95–1.25 kg average BW) were assigned (8 pens/diet; 1 pigs/pen) into three dietary treatments and fed with a basic formula (BF), standard premature infant formula (SF) and hydrolyzed protein formula (HF) respectively, for 7 d. After the piglets sacrifice, growth performance, amino acid metabolism and intestinal morphology were assessed. 16S rRNA sequencing and microbial metabolic phenotypes analyzed the effects of different formula treatments on intestinal flora structure of LBW piglets.

**Results:**

The HF diet reduced the rates of diarrhea and milk vomiting were reduced by 20.44% (*p* > 0.05) and 58.44% (*p* > 0.05), and decreased the crypt depth in the ileum while increasing the ratio of villus height/crypt depth and the mRNA expressions of ^y+^LAT1 and ^b0,+^AT in the ileum (*p* < 0.05). HF increased the final body weight, serum Thr and essential amino acid contents, and CAT2 and ^b0,+^AT mRNA expressions in ileal mucosa compared with the SF diet (*p* < 0.05). Microbiota sequencing results showed that the colonic microbial richness indices (Chao1, ACE, and observed species), the diversity indices (Shannon and Simpson), and the phyla Actinobacteriota, unidentified_Bacteria, Acidobacteriota and Actinobacteria, the genus Rubrobacter and RB41 were reduced (*p* < 0.05) in SF and HF groups. Microbial metabolic phenotypes analysis showed a reduction in the richness of biofilm-forming bacteria (*p* < 0.05).

**Conclusion:**

In summary, hydrolyzed protein formula had better nutrition and tolerance in LBW suckling piglets by improving amino acid transport and intestinal development, and regulating gut microbial communities.

## 1 Introduction

With the advancement of medical technology, the survival rates of premature (<37 weeks gestation) and low birth weight (LBW) infants have significantly increased. However, premature infants are still at a higher risk of developing complications due to their low birth weight and inadequate nutritional intake. Complications such as necrotizing enterocolitis, respiratory distress syndrome, and nutritional absorption difficulties are common among premature infants and unfortunately can lead to high mortality rates (1). The early nutritional supply of premature infants, especially during hospitalization in the Neonatal Intensive Care Unit (NICU), plays a crucial role in their ability to catch up with extrauterine growth and development, achieve catch-up growth, and later nervous system development (2). Therefore, appropriate feeding and formula nutrition are crucial for improving the growth and development of premature and LBW infants.

Breast-feeding is the preferred choice for premature infants, but formula feeding and intravenous infusion are often necessary to meet the infant’s nutritional needs when breast-feeding is not available (3). Breast milk is considered the gold standard of infant formula, and the development of infant formula aims to closely mimic its composition. Utilizing protein technology represents a promising approach to enhancing premature infant formula. Previous studies have demonstrated that hydrolyzed protein formula can reduce feeding intolerance and other complications in premature infants, while also improving gastrointestinal tolerance (4). Hydrolyzed protein formula retains the basic nutritional components such as protein, fat, carbohydrates, vitamins and minerals in the process of hydrolyzing large molecular proteins into small molecules (5). And hydrolyzed whey protein formula can stimulate the secretion of gastrin and motilin, leading to faster gastric emptying and increased peristalsis in the gastrointestinal tract. This formula is effective in treating milk protein allergies, feeding intolerance, and other related diseases (6). For some infants and young children with special conditions such as gastrointestinal disorders, premature or low birth weight infants, hydrolyzed protein formula can provide suitable nutritional support.

Conducting randomized clinical trials with preterm and LBW infants is complex, and using limited human colostrum for animal studies is equally challenging. During the third trimester, most tissues and organs in human babies reach relative maturity. However, preterm birth mainly affects the muscles, central nervous system, and intestinal system (7). Piglets share similarities with humans in terms of growth and development; both species depend heavily on breast milk for survival and growth during early life stages. As a model animal, piglets offer many advantages for studying various tissue and organ states in early life. LBW suckling piglet model has a higher incidence of intestinal issues, insufficient enzyme secretion, low hormone receptors, and developmental delays (8). LBW suckling piglets exhibit respiratory, nutritional, immune, and metabolic impairments that are similar to those observed in human preterm/LBW infants (9). Therefore, LBW suckling piglets are excellent experimental animal models for evaluating the nutrition and metabolism of preterm infants. In particular, studies on nutrition absorption and metabolism provide a unique opportunity to investigate and understand the pathogenesis, prevention, and treatment of premature/LBW pigs. The advantages of using LBW piglets include their ease of handling/operation and their similarity to human infants in terms of gastrointestinal characteristics. These studies are beneficial as they allow for easy management and operation while providing insights into gastrointestinal characteristics shared with human infants.

The objective of this study was to investigate whether hydrolyzed protein formula could provide benefits in terms of growing development, feeding intolerance and nutritional status for premature/LBW infants. To achieve this, we investigated the impacts of hydrolyzed protein formula on growth, amino acid metabolism, intestinal structure, microbiology, and function in LBW suckling piglets as a model of human premature/LBW infants.

## 2 Materials and methods

### 2.1 Ethics statement

All experimental programs and care standards were approved by the Animal Care Committee and Use Committee of the Institute of Subtropical Agriculture, Chinese Academy of Science (Changsha, CAS20220309).

### 2.2 Experimental animals and design

A total of 24 LBW male piglets (Duroc× Large White × Landrace, 3 days old, 0.95–1.25 kg BW) were allocated randomly into three dietary treatments and housed in individual crates. After a 3-day adaptive phase, piglets were fed with basal infant formula (BF), standard premature infant formula (SF), and hydrolyzed protein formula (HF) for 7 days, respectively. Each group contained eight replicates with one piglet in each replicate. The piglets were individually hand-fed from bottles with soft teats from 8:00 to 23:00 h (6 feeds per day) throughout the study. All piglets were housed in a temperature-controlled room maintained at 28 ± 2°C with a 16:8 h light: dark cycle. A target intake for piglets receiving milk is 42 g DM/kg BW per day (10). The 0.95–1.25 kg BW of LBW piglets were selected on the basis that they were weighed less than 20% of the average weight at the neonatal period and the third day. The standard premature infant formula, as a positive control milk powder, is a good formula for the nutrition of premature infants. The ingredient composition of diets required for the study is shown in Table 1. All formula materials for all groups are provided by the Inner Mongolia Yili Industrial Group, Co. Ltd. (Beijing, China).

**Table 1:** Ingredient composition of diets required for the study (g/100 g).

Component^1^	BF	SF	HF
Energy, kJ	2097	2084	2,110
Protein, g	14.62	14.4	14.56
Carbohydrate, g	51.96	51.82	53.8
Fat, g	25.83	25.9	25.63

BF, basal infant formula; SF, standard premature infant formula; HF, hydrolyzed protein formula.

1 All values were measured. Gross energy was measured by an Isothermal Automatic Heat Meter (5E-AC8018, Kaide Automatic Equipment Changsha Co., Ltd., Changsha, China). Crude protein (method GB/T 6432–1994), Fat (method GB/T 14772–2008), carbohydrate (method GB/T 5009.7–2016) were determined.

### 2.3 Sample collection

At the end of the experiment, all pigs were weighed, anesthetized with sodium pentobarbital, and then sacrificed after fasting for 12 h. Blood samples were obtained from the principal vein and collected in 10 mL centrifuged tubes. The tubes were left to stand quietly at room temperature for an hour and then centrifuged at 3500 × g for 10 min at 4°C to obtain serum samples. After dissecting piglets, the heart, liver, spleen, and kidney were quickly removed and weighed. Approximately 2 cm segments of the duodenum, jejunum, and ileum were cut and stored in 4% phosphate-buffered paraformaldehyde (pH 7.6) for histological analysis. After being flushed with 0.9% ice-cold physiological saline solution, the ileal mucosa was collected promptly and frozen in liquid nitrogen, then stored at −80°C for molecular analysis. Colonic contents were collected in sterile tubes and stored at −80°C for the determination of microbiota composition.

### 2.4 Assessment of growth performance and body condition

Throughout the experimental stage, the initial and final body weights, and feed intake were measured and recorded. The study involved the determination of the average daily weight gain (ADG) and Feed/Gain ratio. Additionally, body length was measured from the midpoint of the two ears to the tail root, while the head girth was measured as the natural length of a loop from the ears to the chin. The diarrhea rate was calculated as the number of piglets with diarrhea/ (total numbers of piglets × days) × 100. The milk spitting rate was calculated as the number of piglets with spotting/ (total numbers of piglets × days) × 100.

### 2.5 Detection of serum total protein and free amino acid contents

The total protein content of serum was measured by the BCA Protein Detection Kit (Beyotime, Shanghai, China) according to the kit instructions. The amino acid content of serum was determined by an L-8900 automatic amino acid analyzer (Hitachi8900, Tokyo, Japan). Serum samples (1 mL) were extracted and purified in 8% sodium sulfosalicylate solution (V: V) for 24 h at 4°C. After being centrifuged at 8000 × g for 10 min, the supernatant was filtered using a 0.22 μm filter. Then, it was analyzed using the ninhydrin post-column derivatization method to measure amino acid content (11).

### 2.6 Real-time quantitative analysis

Total RNA of frozen ileal mucosa samples were extracted by the Trizol (Invitrogen, Carlsbad, CA, USA) method. RNA concentration was measured using the nucleic acid concentration detector (Eppendorf AG, Hamburg, Germany). All concentrations of RNA were adjusted to 250 ng/μL and reverse transcribed into double-strand cDNA according to the instructions of the First-Strand cDNA Synthesis Kit (Takara, Otsu, Japan). Obtained cDNA production was used for Real-time PCR to measure the expression of genes related to lipid metabolism and endoplasmic reticulum stress. The extraction process of Real-time PCR was followed by the description of previous study (12). Real-time quantitative PCR was performed by SYBR Green Master Mix reagent (Takara, Otsu, Japan) in a 10 μL reflection system and analyzed by the LightCycler^®^ 480 Real-Time PCR System (Roche, Switzerland, Germany). Relative Ct value (2^−ΔΔCt^) method was used to determine the fold changes in target genes. The housekeeping gene and target gene primers are shown in Table 2.

**Table 2:** Real-time quantitative PCR primer sequences.

Gene	Accession no.	Primer, 5′–3′	Size (bp)
GAPDH	NM_001206359.1	F: CCAGGGCTGCTTTTAACTCTG R:GTGGGTGGAATCATACTGGAACAT	100
ASCT2	XM_003127238.1	F: GATTGTGGAGATGGAGGATGTGG R: TGCGAGTGAAGAGGAAGTAGATGA	149
CAT1	NM_001012613.1	F: TCTGGTCCTGGGCTTCATAA R: ACCTTCGTGGCATTGTTCAG	123
CAT2	NM_001110420.1	F: ACAACTGGCGAAGAAGTCCG R: CTGCCGAGACCCCAAAATAG	100
^y +^LAT1	NM_001110421.1	F: GAGTGCCAGAACACAAACGA R: TCCTCCATCTTCCAAATCCA	116
LAT2	XM_011978238.1	F: CACCATTCCCTGGCTACTCT R: TCCTACCACTGCCTGACAAA	185
^b 0,+^AT	NM_001110171.1	F: GCCTATCAAGGTGCCCATC R: AGCGGACGAACAGGAAGTAA	155
EAAC1	NM_001164649.1	F:GGCACCGCACTCTACGAAGCA R: GCCCACGGCACTTAGCACGA	177
PepT1	NM_214347.1	F: CATCGCCATACCCTTCTG R: TTCCCATCCATCGTGACATT	143

GAPDH, glyceraldehyde-3-phosphate dehydrogenase; ASCT2, autologous stem cell transplantion 2; CAT, chloramphenicol acetyltransferase; ^y+^LAT1, ^y+^-L-type amino acid transporter 1; LAT2, L-type amino acid transporter 2; ^b0,+^AT, b0,+ amino acid transporter; EAAC1, excitafory amino acid cartier 1; PepT1, peptide-transporters 1.

### 2.7 Determination of intestinal morphology

The evaluation of intestinal histomorphological changes was analyzed according to the method of Histological hematoxylin–eosin (HE) staining (13). Three cross-sections were selected from each intestinal middle segment of each piglet, and embedded in paraffin, stained with HE. Under the computer-assisted microscopy (Leica DMI3000B microscopy, Switzerland, Germany), 10 intact, well-oriented crypt-villus units were selected and measured at 100× magnification for analyzing morphological indices and calculating the ratio of villus height to crypt depth (VH/CD).

### 2.8 Intestinal microbial DNA extraction and 16S rRNA sequencing

About 5 g of mix colonic contents for each piglets (*n* = 6 per group) were prepared for bacterial genomic DNA extraction and 16S rRNA sequencing. According to the manufacturer’s instructions, the colonic bacterial genomic DNA was extracted using a PowerFecal DNA Isolation kit (Megan, Guangzhou, China). The purity and concentration of all extracted DNA were determined by 0.80% agarose gel electrophoresis and the nucleic acid concentration detector (Eppendorf AG, Hamburg, Germany). The PCR reaction procedure and components were completed by the Novogene Bioinformatics Technology Co., Ltd. The V4-V5 region of the bacterial 16S rRNA genes was amplified by the PCR-sequence specific primer (F: 5’-ACTCCTACGGGAGGCAGCA-3′, R: 5’-GGACTACTCGGGTATCTAAT-3′). Sequencing libraries were generated by a TruSeq^®^ DNA PCR-Free Kit (Illumina, United States). The quality assessment of the library was carried out using the Qubit@ 2.0 Fluorometer (Thermo Scientific) and Agilent Bioanalyzer 2,100 system. The library sequencing was done on the Illumina MiSeq 2 × 250 platform, which generated 250 bp paired-end reads (14). FLASH is a tool that merges paired-end reads, identifies samples with barcodes (15). The 16S rRNA gene sequence was submitted to the NCBI Sequence Read Archive database under accession number PRJNA1071324.

### 2.9 Microbiological function and phenotypic prediction

The PICRUSt software package predicted functional profiles mainly map 16 rRNA gene sequence reads for KEGG-type pathway prediction. The linearly combine the precomputed functional profiles of the KEGG organisms used Normalized Taxonomic abundances for predicting the microbial functional profile. PICRUSt’s forecasts are based on the Greengenes database and metagenomic 16S rRNA data (16). In addition, Bugbase mainly makes phenotypic prediction and mapps files based on 16S RNA datasets (17). Phenotypes include Gram Positive, Gram Negative, Biofilm Forming, Pathogenic, and Mobile Element Containing, Oxygen utilization (including Aerobic, Anaerobic, facultatively anaerobic) and Oxidative Stress tolerance.

### 2.10 Statistical analysis

Data were expressed as Mean ± SEM, and analyzed by analyses of One-way ANOVA and Duncan multiple comparison using IBM SPSS 23.0 software (SPSS Inc., Chicago, IL, United States). Probability values with *p* ≤ 0.05 were considered statistically significant, whereas values with 0.05 < *p* ≤ 0.10 were declared as showing significant trends.

## 3 Results

### 3.1 HF improves growth performance in LBW piglets

The effects of different formula treatments on the growth performance and body condition in LBW suckling piglets are presented in Table 3. Compared to BF and SF diets, the HF diet had a significant trend of an increased ADG (*p* = 0.080), and the feed/ weight ratio decreased by 38.15% (*p* > 0.05) and 31.6% (*p* > 0.05), respectively. Compared to the SF treatment, the final body weight was significantly increased (*p* < 0.05) in the HF treatment, and rates of diarrhea and milk vomiting were reduced by 20.44% (*p* > 0.05) and 58.44% (*p* > 0.05), respectively. The initial body weight of piglets had no significant differences among the treatments (*p* > 0.05).

**Table 3:** Effects of different formula treatments on the growth performance and body condition in LBW suckling piglets.

Item	Group^1^			*p*-value
	BF	SF	HF	
Initial body weight, kg	1.19 ± 0.02	1.17 ± 0.02	1.18 ± 0.02	0.986
Final body weight, kg	1.47 ± 0.02^ab^	1.42 ± 0.04^a^	1.54 ± 0.02^b^	0.043
ADG, g/d	39.17 ± 3.38	38.33 ± 5.27	51.4 ± 3.77	0.080
Feed/weight ratio	1.05 ± 0.15	1.00 ± 0.08	0.76 ± 0.04	0.122
Body length, cm	29.22 ± 0.51	29.07 ± 0.45	29.87 ± 0.84	0.639
Head girth, cm	23.02 ± 0.34	22.78 ± 0.27	22.52 ± 0.39	0.592
Diarrhea rate, %	11.54 ± 6.66	16.92 ± 6.15	13.46 ± 5.77	0.822
Milk spitting rate, %	0	4.62 ± 1.88	1.92 ± 1.92	0.173

1 BF, basal infant formula; SF, standard premature infant formula; HF, hydrolyzed protein formula.

Data are expressed as means ± SEM (*n* = 8). Means within a row with different superscripts are significantly different (*P* < 0.05).

### 3.2 HF has no effect on organ weight in LBW piglets

The organ weight results of LBW suckling piglets are listed in Table 4. Compared to both BF and SF diets, the HF diet did not show a significant difference in organ weight and intestinal indexes of LBW suckling piglets (*p* > 0.05).

**Table 4:** Effects of different formula treatments on the organ weight in LBW suckling piglets.

Item	Group^1^			*P*-value
	BF	SF	HF	
Heart, g	10.46 ± 0.62	10.32 ± 0.52	11.4 ± 0.39	0.302
Liver, g	56.1 ± 2.86	52.32 ± 3.92	57.9 ± 5.77	0.659
Spleen, g	2.27 ± 0.25	2.57 ± 0.26	3.12 ± 0.33	0.137
Lung, g	18.65 ± 1.08	18.3 ± 1.2	17.37 ± 0.92	0.688
Kidney, g	9.33 ± 0.47	9.3 ± 0.67	10.53 ± 0.39	0.200
Head, g	31.73 ± 1.12	30.79 ± 0.45	31.65 ± 1.09	0.741
Small intestinal, g	69.34 ± 5.45	66.62 ± 4.51	58.67 ± 11.15	0.600
Small intestine length, cm	370.67 ± 20.39	385.17 ± 17.88	414.5 ± 36.85	0.505
Mean intestinal weight, g/cm	0.19 ± 0.02	0.18 ± 0.01	0.18 ± 0.01	0.739

1 BF, basal infant formula; SF, standard premature infant formula; HF, hydrolyzed protein formula.

Data are expressed as means ± SEM (*n* = 8). Means within a row with different superscripts are significantly different (*P* < 0.05).

### 3.3 HF increases serum amino acid contents in LBW piglets

To evaluate the effects of different formula treatments on serum amino acid content in LBW suckling piglets were analyzed using an automatic amino acid analyzer (Table 5). Compared to SF treatment, both BF and HF increased the contents of Thr and essential amino acid (EAA, *p* < 0.05). The SF group had a higher Val content compared with the BF and HF treatments (*p* < 0.05).

**Table 5:** Effects of different formula treatments on serum total protein and amino acid contents in LBW suckling piglets.

Item^2^	Group^1^			*p*-value
	BF	SF	HF	
Total protein, g/L	47.23 ± 2.41	47.43 ± 2.57	48.17 ± 3.34	0.970
Essential amino acids, μg/mL				
Thr	25.83 ± 2.83^b^	8.69 ± 1.91^a^	33.46 ± 5.09^b^	0.001
Val	9.14 ± 0.78^b^	6.74 ± 0.62^a^	7.15 ± 0.54^a^	0.044
Ile	4.25 ± 0.28	3.45 ± 0.4	3.53 ± 0.33	0.226
Leu	6.87 ± 0.56	5.76 ± 0.55	5.47 ± 0.39	0.153
Met	4.05 ± 0.68	3.94 ± 0.46	4.91 ± 0.44	0.401
Phe	5.79 ± 0.67	6.24 ± 0.63	6.06 ± 0.36	0.856
Lys	9.59 ± 1.43	11.21 ± 1.86	9.13 ± 1.42	0.630
His	4.35 ± 0.49^a^	7.07 ± 1.01^b^	4.09 ± 0.57^a^	0.020
Arg	8.83 ± 0.44	7.98 ± 0.75	8.96 ± 0.75	0.544
Non-essential amino acids, μg/mL				
Asp	2.42 ± 0.55	2.33 ± 0.33	2.37 ± 0.24	0.988
Ser	9.68 ± 1.2	9.51 ± 1.03	10.95 ± 0.89	0.583
Glu	13.54 ± 1.99	14.2 ± 1.82	12.2 ± 1.03	0.694
Gly	35.78 ± 5.01	42.12 ± 4.37	38.4 ± 4.75	0.643
Ala	21.6 ± 2.03	20.06 ± 2.29	18.81 ± 1.51	0.614
Cys	2.58 ± 0.35	2.37 ± 0.37	2.72 ± 0.35	0.780
Pro	9.21 ± 1.31	11.1 ± 1.2	9.9 ± 1.43	0.603
Tyr	3.24 ± 0.34	4.06 ± 0.39	3.19 ± 0.25	0.149
EAA	78.71 ± 3.57^b^	61.08 ± 5.54^a^	82.75 ± 6.68^b^	0.029
TAA	172.79 ± 11.85	162.64 ± 15.11	179.83 ± 9.99	0.629

1 BF, basal infant formula; SF, standard premature infant formula; HF, hydrolyzed protein formula.

2 EAA, essential amino acid; TAA, total amino acid.

Data are expressed as means ± SEM (*n* = 8). Means within a row with different superscripts are significantly different (*P* < 0.05).

### 3.4 HF promotes intestinal morphological development in LBW piglets

**Figure 1:**
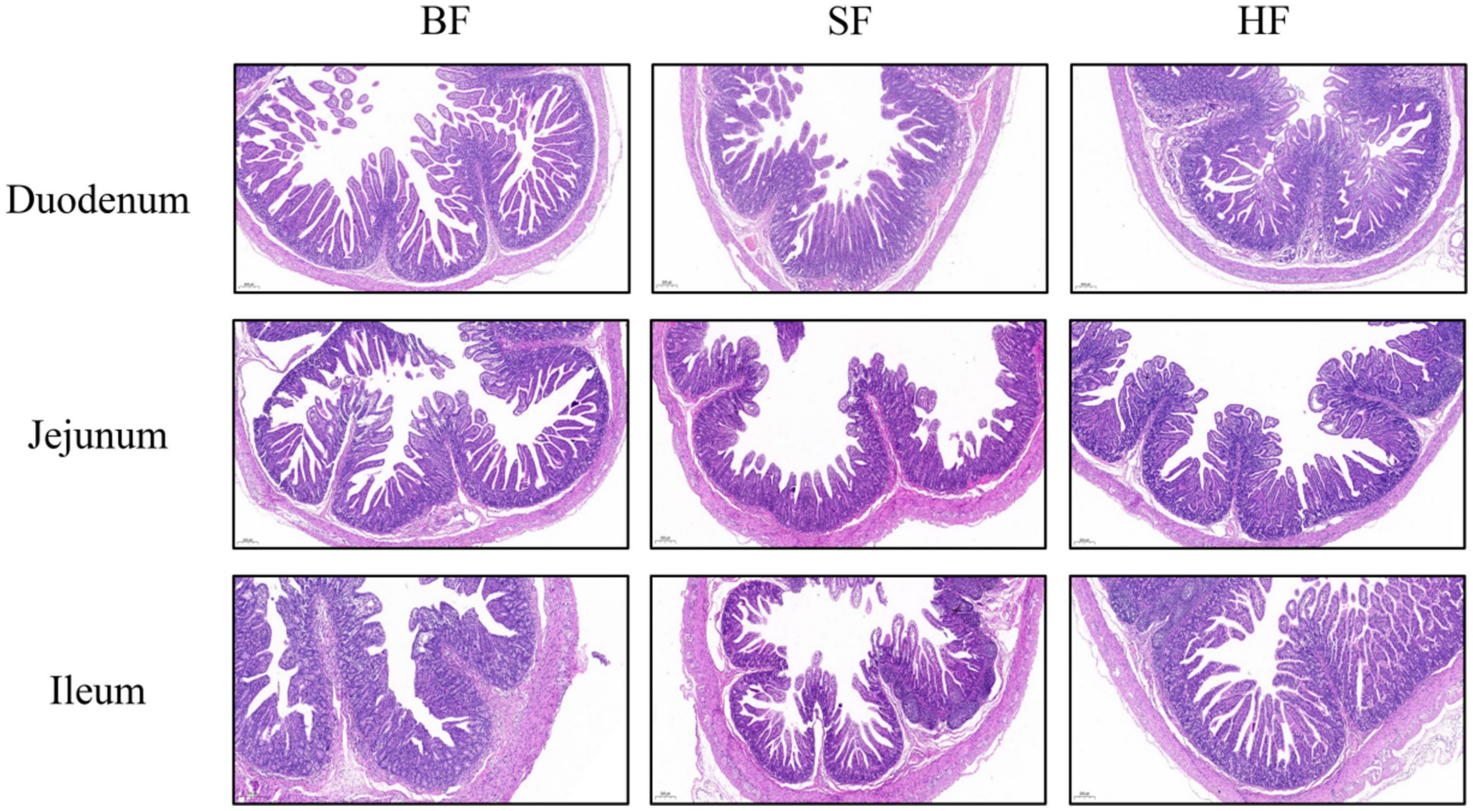
The intestinal morphology was histologically analyzed by hematoxylin and eosin (HE, 200 μm). (A) Duodenum, (B) Jejunum, (C) Ileum; BF, basal infant formula; SF, standard premature infant formula; HF, hydrolyzed protein formula.

The intestinal morphology results of LBW suckling piglets are listed in Table 6 and Figure 1. Compared with the BF group, SF and HF groups significantly decreased (*p* < 0.05) the crypt depth in the jejunum, the HF group decreased (*p* < 0.05) the crypt depth and increased the ratio of villus height/crypt depth (*p* < 0.05) in the ileum. In the jejunum, compared to the BF and HF diets, the SF diet increased (*p* < 0.05) the ratio of villus height/crypt depth.

**Table 6:** Effects of different formula treatments on intestinal morphology in LBW suckling piglets.

Item	Group^1^			*P*-value
	BF	SF	HF	
Duodenum				
Villus height, μm	252.8 ± 9.12	235.19 ± 7.1	236.13 ± 6.43	0.191
Crypt depth, μm	90.77 ± 3.56	93.21 ± 3.74	90.11 ± 3.34	0.807
Villus height/Crypt depth	2.96 ± 0.15	2.7 ± 0.13	2.74 ± 0.09	0.268
Jejunum				
Villus height, μm	258.09 ± 5.67	251.29 ± 9.06	248 ± 7.56	0.634
Crypt depth, μm	84.22 ± 2.32^b^	71.49 ± 3.13^a^	75.49 ± 2.04^a^	0.002
Villus height/Crypt depth	3.2 ± 0.12^a^	3.8 ± 0.18^b^	3.37 ± 0.12^a^	0.012
Ileum				
Villus height, μm	206.47 ± 12.54	218.99 ± 10.67	213.05 ± 6.89	0.692
Crypt depth, μm	81.32 ± 1.83^b^	77.87 ± 2.82^ab^	71.85 ± 2.96^a^	0.035
Villus height/Crypt depth	2.61 ± 0.17^a^	2.94 ± 0.15^ab^	3.24 ± 0.2^b^	0.041

1 BF, basal infant formula; SF, standard premature infant formula; HF, hydrolyzed protein formula.

Data are expressed as means ± SEM (*n* = 8). Means within a row with different superscripts are significantly different (*P* < 0.05).

### 3.5 HF up-regulates the expression of genes associated with amino acid transporters in the ileum of LBW piglets

**Figure 2:**
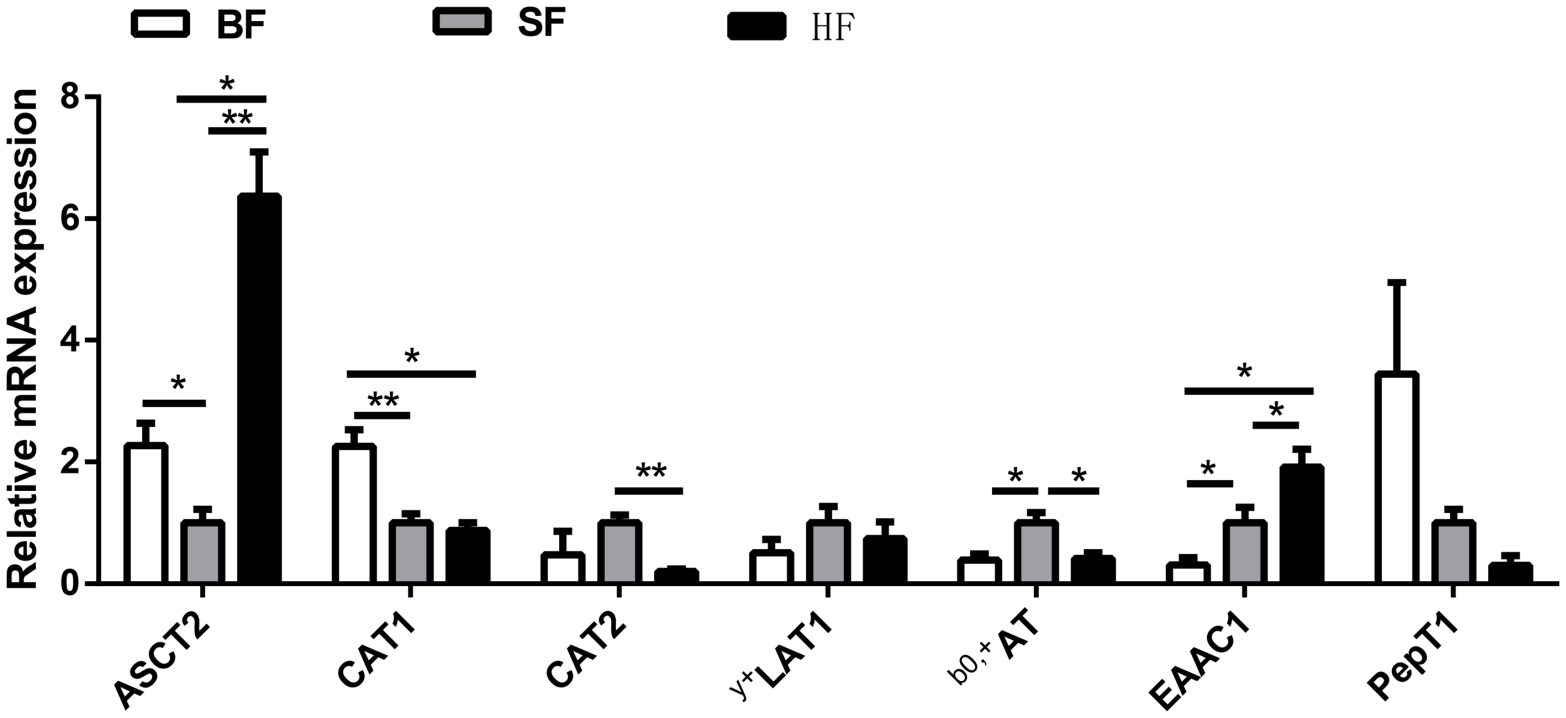
Effect of different formula treatments on gene expression levels associated with the amino acid transporters in ileal mucosa. BF, basal infant formula; SF, standard premature infant formula; HF, hydrolyzed protein formula. Data are expressed as means ± SEM (*n* = 6). *p < 0.05 and **p < 0.01.

Analysis of the mRNA expression levels of amino acid transporters in ileal mucosa revealed differences among different formula treatments (Figure 2). Compared to the BF diet, the HF diet significantly down-regulated (*p* < 0.05) the mRNA expression of ASCT2 and CAT1, while up-regulated (*p* < 0.05) the mRNA expression of ^y+^LAT1 and ^b0,+^AT. HF group had lower (*p* < 0.05) mRNA expressions of ASCT2 and EAAC1, and higher (*p* < 0.05) mRNA expressions of CAT2 and ^b0,+^AT compared to the SF group.

### 3.6 HF improves colonic microbiota diversity and composition in LBW piglets

**Figure 3:**
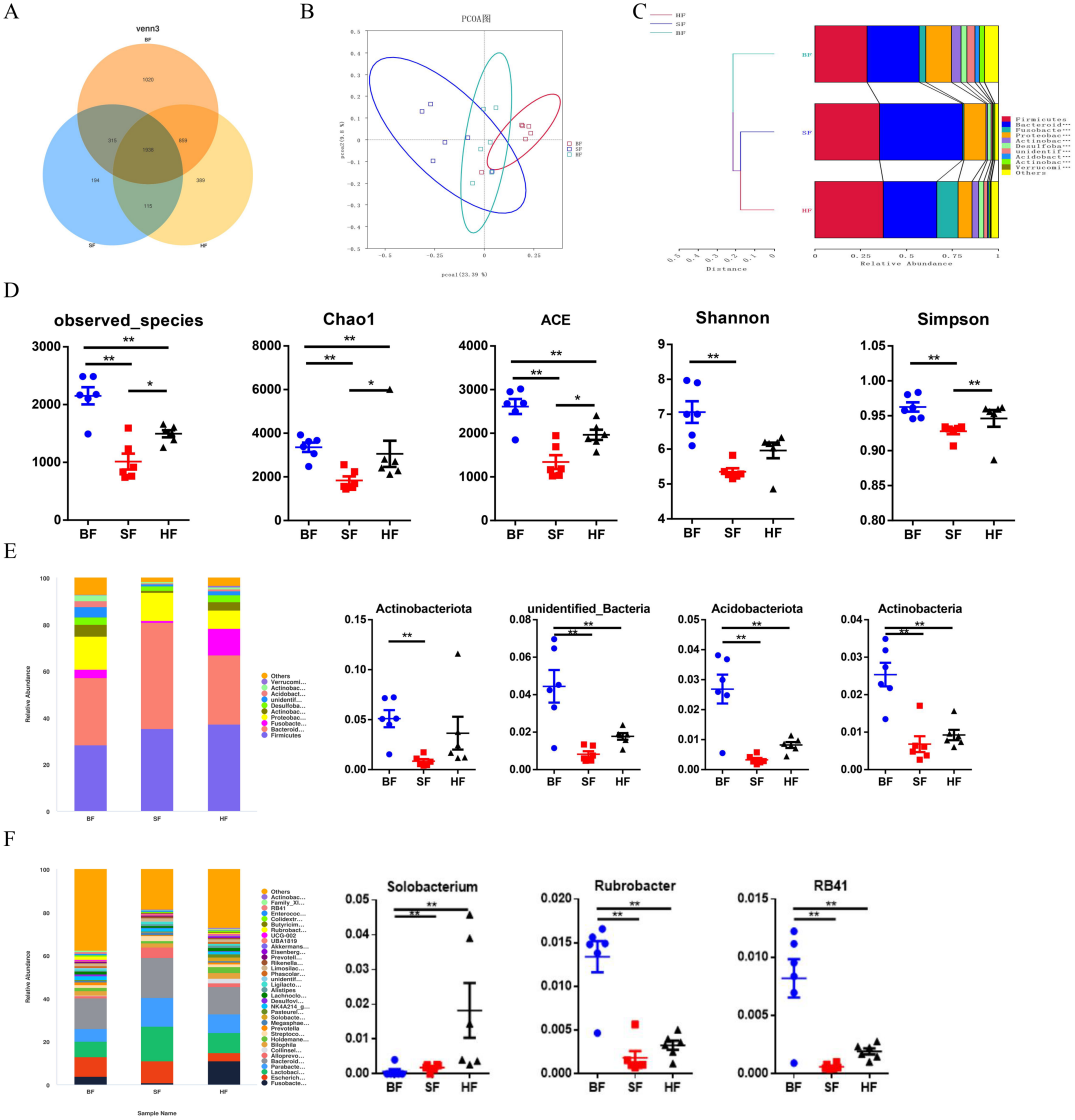
Effect of different formula treatments on colonic microbiota diversity and composition in pigs. (A) A Venn diagram illustrating the overlaps of OTUs in the gut microbiota. (B) Principal coordinate analysis (PCoA). (D) Non-metric multidimensional scaling (NMDS) analysis. (C) Unweighted unifrac cluster tree based on Unweighted Pair-group Method with Arithmetic Mean (UPGMA) analysis. (D) The microbial alpha diversity index (Observed-species, Chao1, Shannon, Simpson, ACE) were calculated using the mother program. (E) Relative contribution of the top 10 phylum in each group (left) and the relative abundance of significantly different microorganisms (right). (F) The relative contribution of the top 35 genera in each group (left) and the relative abundance of significantly different microorganisms (right). BF, basal infant formula; SF, standard premature infant formula; HF, hydrolyzed protein formula. Data are expressed as means ± SEM (*n* = 6). *p < 0.05, **p < 0.01, and ***p < 0.001.

To assess the colonic microbiota composition in response to different formula treatments, colonic contents of LBW suckling piglets were collected for metagenomic sequencing. The variations in the degree of overlap richness among treatments were visually represented using a Venn diagram (Figure 3A). This analysis showed that BF, SF, and HF contained 1,020, 194, and 389 unique OTUs, respectively. The principal coordinate analysis (PCoA) (Figure 3B) revealed a significant microbial distinction among the three treatments. An unweighted Unifrac cluster tree, generated using the unweighted pair-group method with arithmetic mean (UPGMA) analysis, demonstrated the similarity and phylogeny of all observed samples at the phylum level (Figure 3C). Firmicutes, Bacteroidetes, Fusobacteriota, and Proteobacteria were identified as the predominant bacteria in piglets’ colonic microbiota. As shown in Figure 3D, compared with the BF group, HF significantly decreased the microbial richness indices (Chao1, ACE, and observed species) (*p* < 0.05) in the gut microbiota of piglets. Compared with the SF group, HF significantly increased (*p* < 0.05) Chao1, ACE, observed species, and diversity indices (Simpson). The phylum level analysis, as depicted in Figure 3E, revealed that the relative abundances of Actinobacteriota, unidentified_Bacteria, Acidobacteriota, and Actinobacteria were significantly reduced (*p* < 0.05) with dietary supplementation of SF and HF diets. Compared to the BF group, the SF group decreased (*p* < 0.05) the relative abundance of Actinobacteriota. In the genus level (Figure 3F), compared with the BF diet, SF and HF diets significantly decreased the relative abundances of Rubrobacter and RB41 (*p* < 0.05). HF group had the highest relative abundance of Solobacterium (*p* < 0.05) than BF and SF groups.

### 3.7 Metabolic functions and phenotypes of colonic microbiota

**Figure 4:**
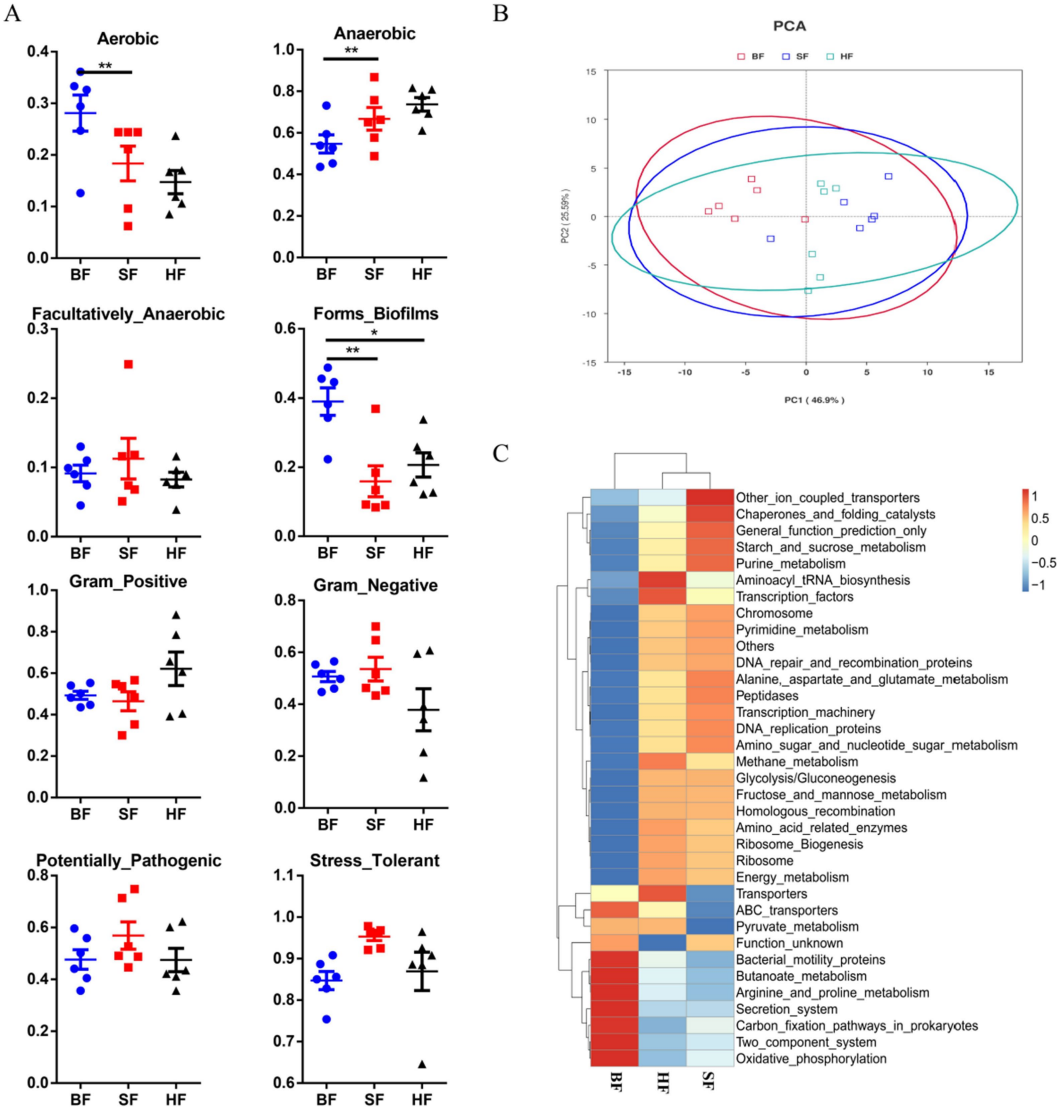
Dietary supplementation with different formulas altered the metabolic functions and phenotypes of colonic microbiota. (A) The metabolic phenotype predictions were compared using BugBase online (https://bugbase.cs.umn.edu/). (B) Principal components analysis (PCA) was used to analyze the functional profiles in the gut microbiota. (C) A heatmap tree was created based on different metabolism-related pathways at KEGG level 3. The relative abundances of discrete phenotypes were performed using pair-wise Mann–Whitney U tests. BF, basal infant formula; SF, standard premature infant formula; HF, hydrolyzed protein formula. Data are expressed as means ± SEM (*n* = 6). *p < 0.05 and **p < 0.01.

As depicted in Figure 4A, Bugbase results showed that HF and SF diets significantly decreased (*p* < 0.05) the aerobic bacterial richness and biofilm-forming of colonic microbiota compared to the BF diet. SF treatment had a higher anaerobic bacterial richness (*p* < 0.05) in colonic microbiota than the BF group.

PICRUSt was used to assess the impact of different formula treatments on the metabolic functions of gut microbiota, based on KEGG annotations. Principal component analysis (PCA) was employed to separated microbiota metabolic functions in BF, SF, and HF groups (Figure 4B). At level 3, microbial metabolism is associated with KEGG pathways, as depicted in Figure 4C. Results showed that compared with the BF group, HF and SF groups had higher flora abundances on energy metabolism, amino acid-related enzymes, pyrimidine, purine metabolism, peptidases, and starch_and_sucrose_metabolism, and lower flora abundances on bacterial motility proteins, oxidative phosphorylation, and carbon fixation pathways in prokaryotes.

## 4 Discussion

Milk quality is crucial for the immature gut’s response to first enteral feeding. However, the effects of infant formula on intestinal responses during the first week of life are not yet known, so optimal feeding strategies need to be explored. Hydrolyzed protein formula is a type of powdered milk that has been increasingly used as a feeding option for premature infants (3). In this study, we investigated the effects and molecular mechanisms of hydrolyzed protein formula on the nutritional status and feeding intolerance of premature/LBW infants. Importantly, it was firstly found hydrolyzed protein formula had better nutrition and tolerance in an animal model of LBW suckling piglets. Intervention with hydrolyzed protein formula has the potential to be a new and attractive therapeutic strategy for improving growth for preterm and low birth weight infants.

Premature or LBW infants generally have less developed gastrointestinal peristalsis, digestion, and absorption functions compared to full-term and normal-weight infants. Additionally, due to their smaller stomach volume and insufficient gastric acid secretion capacity, their stomach environment’s pH value is higher, resulting in lower protease and enterocasmin activities. This compromised protein hydrolysis increases the risk of complications related to feeding intolerance (18). Moderately hydrolyzed formula has been reported to promote intestinal absorption and improve nutritional efficiency, which have certain benefits for babies whose intestinal functions are not fully developed or have digestive disorders. Therefore, to evaluate nutrient availability in LBW suckling piglets, we assessed their growth performance and body condition indicators. We observed that the HF diet increased final body weight and ADG while decreasing the feed-to-weight ratio. These results indicate a positive impact on growth performance in LBW suckling piglets. The feed-to-weight ratio serves as a measure of nutrient utilization efficiency within the body. A lower feed/ weight ratio indicates higher nutrient conversion efficiency. Additionally, the HF diet may lead to a decrease in the incidence of diarrhea and milk vomiting, which suggests that hydrolyzed protein formulas may reduce the occurrence of feeding intolerance symptoms. Several studies also demonstrated that hydrolyzed protein formula can better promote extrauterine growth and development of premature infants than common formula (19). Collectively, these data indicated the beneficial effects of hydrolyzed protein formula on growth performance. Its key mechanism may be related to the smaller size of proteins in hydrolyzed protein formula is closer to the size of breast milk proteins, and easier to digest and sorption.

Serum total protein and amino acid indexes are often used to study protein metabolism and evaluate animal amino acid requirements. Importantly, the plasma metabolism profile changes according to dietary manipulations and affects physiological and metabolic activities (20). This present study indicated that HF improves the contents of Val, Thr, and EAA in the serum, while has no influence on total protein level. Notably, no change in serum total protein levels confirmed that the protein of the hydrolyzed protein formula was cleaved into short peptide chains and amino acids. Consistent with previous research, hydrolyzed protein formula produces more amino acids and polypeptides than ordinary formula from gastrointestinal tract hydrolyzed protein (21). Additionally, specific transporters are crucial in transporting amino acids into the cell and responding to amino acid availability (22). Previous studies have found that amino acid transporters undergo changes in the small intestine, skeletal muscle, and other tissues once dietary nutrient content was varied (23, 24). Specialized amino acid transporters, featuring unique substrate specificities and mechanisms, play critical roles in cellular transportation (25). The ASCT2, ^b0, +^AT, ^y+^LAT1, and LAT2 are responsible for transporting neutral amino acids and some small amino acids, including branched-chain amino acids (leucine, isoleucine, and valine), asparagine, and glutamine (26). In addition, Na^+^ independent transporters include ^b0, +^AT, and ^y+^LAT1 (27). The cationic amino acid transporters (CATs) are widely distributed in tissues and responsible for maintaining the homeostasis of ornithine, histidine, lysine and arginine (28). In our study, the HF diet decreased ASCT2 and CAT1 mRNA expression, and increased ^y+^LAT1 and ^b0,+^AT mRNA relative expression compared with the BF diet. HF group had lower mRNA expressions of ASCT2 and EAAC1 and higher mRNA expressions of CAT2 and ^b0,+^AT in the ileum than the SF group. Thus, it is possible to explain that the hydrolyzed protein formula may promote amino acid absorption by increasing the expression of intestinal amino acid transporters, and then increase serum amino acid content.

The enhancement of intestinal morphology and intestinal barrier helps to improve nutrient absorption and intestinal integrity, thus promoting growth and development (29). Intestinal morphology injury, resulting in diarrhea and growth retardation in pigs due to villous atrophy and crypt hyperplasia (30). An increase in villus height/ crypt depth ratio is a crucial indicator of improved intestinal morphology, evaluating intestinal function and absorption capacity (31). The present study found that the HF diet decreased the crypt depth in the jejunum and ileum, and increased the ratio of villus height to crypt depth in the ileum, and the SF diet increased the ratio of villus height/ crypt depth in the jejunum. Therefore, we speculated that SF and HF could enhance the development of intestinal villi and digestive ability of the small intestines. Feng et al. also found that hydrolyzed protein formula could promote the secretion of motilin, thereby enhancing gastrointestinal maturation and tolerance (32). It is suggested that hydrolyzed protein formula can promote intestinal development and reduce diarrhea in premature infants.

The gut microbiome is a critical biomarker involved in evaluation of the effect of specific dietary components on the host. The complex microbial ecosystem plays an important role in the host’s health and disease (33). The current study showed that decreases in the microbial richness, and the abundances of the phyla *Actinobacteriota*, *unidentified_Bacteria*, *Acidobacteriota*, and *Actinobacteria* by HF treatment may explain the beneficial regulation of intestinal microbiota in LBW suckling piglets. Piglets fed the HF diet had a greater microbial diversity, as evidenced by higher Chao1, ACE, observed species, and Simpson indices for gut microbiota. Besides, compared with the BF group, SF and HF groups had similar intestinal flora structures in reducing the phyla *unidentified_Bacteria*, *Acidobacteriota*, and *Actinobacteria*, and the genus *Rubrobacter* and *RB41*. *Actinobacteriota* and *Actinobacteria*, as two central communities, are associated with the decomposition of organic materials (34). Remarkably, *Solobacterium* was increased in the colon of LBW piglets fed the HF diet.

According to microbial function prediction, the results showed that HF increased energy metabolism, amino acid-related enzymes, pyrimidine and purine metabolism, peptidases, starch and sucrose metabolism, and decreased bacterial motility proteins, oxidative phosphorylation, and carbon fixation pathways in prokaryotes. The HF diet may improve the amino acid metabolism and protein synthesis in gut microbial communities. Furthermore, the study revealed the differences in microbial metabolic phenotypes in piglets fed with various formulas. Furthermore, our results confirmed that the reduction of the aerobic bacterial richness and biofilm-forming of colonic microbiota by HF and SF treatments. Previous studies revealed that oxidative stress tolerance and biofilm-formation of microbial communities were associated with inflammation, drug resistance and pathogenesis (35, 36). However, it remains unclear how hydrolyzed protein formula targets key bacteria and metabolites to regulate intestinal healthy, and these metabolic phenotypes’ changes need to further explore the mechanism.

Developing and studying hydrolyzed protein formula for premature and LBW infants has considerable theoretical, economic value, and significant social benefit. Breast milk is the most ideal food for infants, and future research will continue to explore how to make the nutritional composition and structure of hydrolyzed protein formula more similar to breast milk. In particular, we will in-depth study the proportion and mechanism of action of various proteins, fats, carbohydrates, probiotics and other components in breast milk to optimize the formula of hydrolyzed protein formula for improving its nutritional value and adaptability.

## 5 Conclusion

In conclusion, hydrolyzed protein formula had better nutrition and tolerance in LBW suckling piglets, as demonstrated by improving amino acid transport, intestinal development, and regulating gut microbial communities. Hydrolyzed protein formula increased serum EAA content, up-regulated CAT2, ^y+^LAT1 and ^b0,+^AT, improved intestinal villus morphology, and F/G ratio. Furthermore, apart from reducing the richness indices, the use of hydrolyzed protein formula has been found to significantly enhance the microbial metabolic phenotypes and functions in piglets, thereby improving their intestinal status and growth potential. This study provides theoretical evidence to support the use of hydrolyzed protein formula in premature and LBW infants to promote their growth.

## Data Availability

The datasets presented in this study can be found in online repositories. The names of the repository/repositories and accession number(s) can be found in the article/supplementary material.
