# Correction: Hydrolyzed protein formula improves the nutritional tolerance by increasing intestinal development and altering cecal microbiota in low-birth-weight piglets

**DOI:** 10.3389/fnut.2025.1755514

**Published:** 2026-01-12

**Authors:** Miaomiao Bai, Hongnan Liu, Yalu Yan, Sufang Duan, Ignatius Man-Yau Szeto, Jian He, Jinjie Hu, Yawei Fu, Kang Xu, Xia Xiong

**Affiliations:** 1 Laboratory of Animal Nutritional Physiology and Metabolic Process, Key Laboratory of Agro-ecological Processes in Subtropical Region, Institute of Subtropical Agriculture, Chinese Academy of Sciences, Changsha, Hunan, China; 2 Inner Mongolia Yili Industrial Group, Co. Ltd, Yili Maternal and Infant Nutrition Institute (YMINI), Beijing, China; 3 Inner Mongolia Dairy Technology Research Institute Co. Ltd, Hohhot, China; 4 National Center of Technology Innovation for Dairy, Hohhot, China; 5 College of Animal Science and Technology, Hunan Agricultural University, Changsha, China; 6 Hunan Provincial Key Laboratory of the Traditional Chinese Medicine Agricultural Biogenomics, Changsha Medical University, Hunan, Changsha, China

**Keywords:** prematurity or low birth weight, hydrolyzed protein formula, amino acid metabolism, intestinal development, gut microbiota

There was a mistake in Figure 3 as published. The image of RB41 was duplicated with the image of Rubrobacter. The corrected Figure 3 appears below.

**Figure 3:**
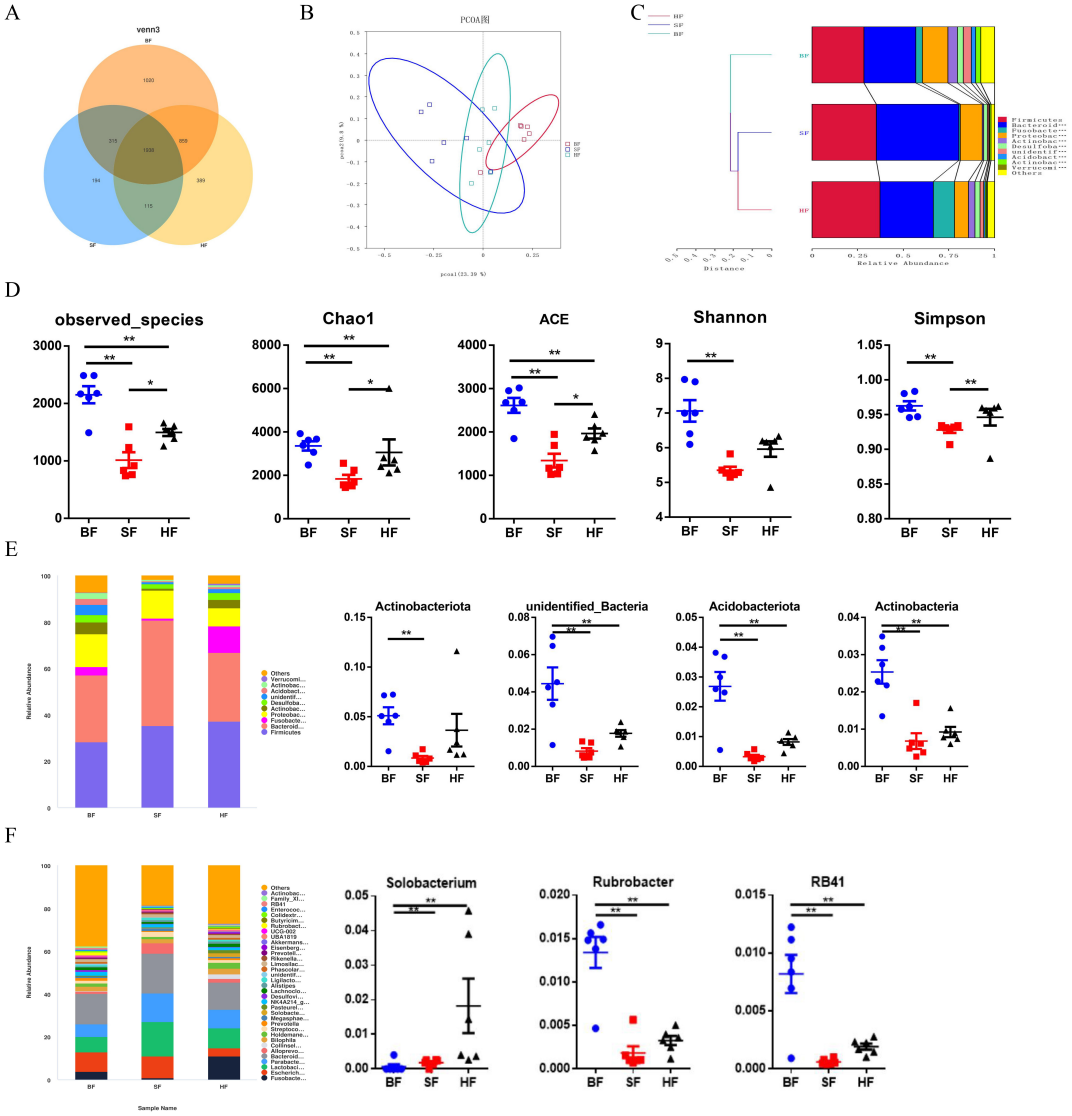
Effect of different formula treatments on colonic microbiota diversity and composition in pigs. **(A)** A Venn diagram illustrating the overlaps of OTUs in the gut microbiota. **(B)** Principal coordinate analysis (PCoA). **(D)** Non-metric multidimensional scaling (NMDS) analysis. **(C)** Unweighted unifrac cluster tree based on Unweighted Pair-group Method with Arithmetic Mean (UPGMA) analysis. **(D)** The microbial alpha diversity index (Observed-species, Chao1, Shannon, Simpson, ACE) were calculated using the mother program. **(E)** Relative contribution of the top 10 phylum in each group (left) and the relative abundance of significantly different microorganisms (right). **(F)** The relative contribution of the top 35 genera in each group (left) and the relative abundance of significantly different microorganisms (right). BF, basal infant formula; SF, standard premature infant formula; HF, hydrolyzed protein formula. Data are expressed as means ± SEM (*n* = 6). ^*^p < 0.05, ^**^p < 0.01, and ^***^p < 0.001.

The original version of this article has been updated.

